# Study on the portal ramification pattern of the right anterior sector of the liver and a unique medial branch (PV8c) of the right anterior portal vein

**DOI:** 10.1002/ags3.12561

**Published:** 2022-03-01

**Authors:** Norihiro Ishii, Norifumi Harimoto, Kimitaka Kogure, Kenichiro Araki, Kei Hagiwara, Mariko Tsukagoshi, Takamichi Igarashi, Akira Watanabe, Norio Kubo, Ken Shirabe

**Affiliations:** ^1^ 12925 Department of General Surgical Science Division of Hepatobiliary and Pancreatic Surgery Graduate School of Medicine Gunma University Gunma Japan; ^2^ 12925 Department of General Surgical Science Graduate School of Medicine Gunma University Gunma Japan; ^3^ Department of Innovative Cancer Immunotherapy Gunma University Graduate School of Medicine Gunma Japan

**Keywords:** anterior sector, caudate lobe, Couinaud segmentation, portal ramification, three‐dimensional imaging

## Abstract

**Aim:**

The concept of Couinaud segmentation is widely used in clinical practice. However, there were no definite anatomical landmarks between segments V and VIII. Therefore, segmentation of the right anterior sector is still controversial. We aimed to investigate the portal segmentation of the right anterior sector using 3D image analysis, and to reveal the existence of the medial branch (PV8c), a unique, characteristic branch of the right anterior portal vein.

**Methods:**

The ramification form and pattern of the tertiary portal branch of the right anterior portal vein were retrospectively analyzed, and the frequency of PV8c was evaluated in 261 patients between January 2016 and June 2020.

**Results:**

The ramification pattern of tertiary portal branches of the right anterior portal vein was classified into four types: craniocaudal, 28.0% of patients; ventrodorsal, 21.8%; trifurcation, 39.5%; and quadfurcation, 5.7%, and each type was further subdivided into six patterns by focusing especially on the caudal branches. The ramification pattern in the remaining 5.0% of the livers did not belong to the above‐mentioned four types. The PV8c branch was identified in 140 of 261 livers (53.6%); the mean proportion of the feeding area of PV8c in the whole liver volume was 3.4%.

**Conclusion:**

Since the ramification pattern of tertiary portal branches of the right anterior portal vein does not necessarily show a single pattern, it is important to confirm the portal vein branching in each case during hepatectomy. This is the first study of the details of PV8c by 3D computed tomography.

## Graphical abstract

The right anterior sector was divided into four types according to the perfusion area of the tertiary portal branch and subclassified into six patterns based on the ramification form. Moreover, this is the first report of the details of PV8c, a unique branch of the right anterior portal vein, by 3D‐CT.

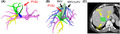

## 1 INTRODUCTION

Liver segmentation based on the portal veins and hepatic veins proposed by Claude Couinaud and Healey and Schroy is useful for determining the location of the tumor and for the treatment of hepatocellular carcinoma (HCC),^1^, ^2^, ^3^ and has been used widely in clinical practice. Furthermore, anatomical resection of HCC has shown a survival benefit compared with nonanatomical resection, because HCC is considered to progress along the feeding portal venous branch.^4^ Thus, an understanding of the portal venous anatomy and its segmentation is important for performing parenchymal‐preserving hepatectomies and avoiding postoperative complications, such as biliary leakage or stenosis.^5^, ^6^, ^7^, ^8^ However, the borders between segments V and VIII or segments VI and VII in the Couinaud classifications are often unclear. Although Couinaud described the inferior limit of segment VIII as a transversal plane through the hepatic hilum,^9^ this is an imaginary line, and there are no anatomical landmarks.^10^

Before the proposal of the Couinaud classification, Hjortsjo proposed that the right anterior sector was divided into ventral and dorsal subsegments by a vertical plane in which hepatic venous branches from the middle or right hepatic vein or an independent hepatic vein.^11^ Furthermore, Mikami in 1956^12^ and Trinh Van‐Minh et al in 1985^13^ divided the right anterior sector into three subsegments using the livers of cadavers in 20 cases and liver casts in 200 cases, which were classified as ventral and dorsal upper areas and a lower area corresponding to the tertiary portal branch trifurcation of the right anterior sector. Although the concept of segmentation of the right anterior sector is still controversial, Couinaud, Hjortsjo, and Trinh Van‐Minh et al's anatomical segmentations are considered to be the three major classifications of the right anterior sector. Historically, anatomical studies of the human liver have been conducted using cadavers,^12^, ^14^ liver casts,^11^, ^13^, ^15^ angiography of radiologic imaging,^16^, ^17^ and, more recently, 3D analysis by computed tomography (CT) images.^5^, ^18^, ^19^, ^20^ Recent reports of 3D‐CT analyses of the right anterior sector have often been reported according to the three aforementioned major classifications.^18^, ^21^ Additionally, Kurimoto et al reported the fourth type (quadfurcation pattern) of the portal branch of the right anterior sector using 3D‐CT image analysis.^5^

On the other hand, Kumon^22^ and Trinh Van‐Minh et al^13^ described a unique, important, and characteristic portal branch of the right anterior sector in a study of the caudate lobe using a liver cast. Kumon described this branch as a medial branch of segment VIII (PV8c), which was defined as follows: “The portal venous branches that ramified from the contralateral side of the segment 5 portal vein root and distributed in the territory surrounded by the roots of the right and middle hepatic veins.^15^” Trinh Van‐Minh et al described the same branch in the figure of the portal venous ramification of the anterior sector and referred to it as “an accessory inconstant portal venous branch.^13^” To our knowledge, although there are reports on the frequency of PV8c using 3D‐CT analysis,^23^, ^24^ there have been no reports on the details of PV8c such as perfusion area, volume, and the interpretations of existence.

This study aimed to investigate the portal segmentation of the right anterior sector, especially focusing on the ramification pattern of tertiary branches, and to reveal the details of PV8c using 3D image analysis.

## 2 METHODS

### 2.1 Ethics statements

This study was conducted in accordance with the institutional guidelines and the Declaration of Helsinki. The Institutional Review Board of Gunma University approved this study protocol (approval number: HS2019‐192).

### 2.2 Study design and population

We retrospectively evaluated patients with suspected hepatobiliary and pancreatic diseases who underwent three‐ or four‐phase contrast multidetector‐row CT (MDCT) between January 2016 and June 2020 at Gunma University Hospital (Maebashi, Japan). Patients with liver cirrhosis and vascular invasion by tumors were excluded from the study.

### 2.3 Imaging procedure

The conditions of contrast‐enhanced CT were as follows. After a series of scans without a contrast agent, 630 mg/kg of nonionic iodine was administered via the antecubital vein at 30 sec with a power injector. Then scanning was performed in the early arterial, late arterial, and portal phases (25, 35, and 60 sec after contrast injection, respectively). In all phases, the slice thickness was 0.625 mm. The MDCT datasets were transferred to a workstation for 3D image analysis using software (Synapse Vincent; Fujifilm, Tokyo, Japan). The liver parenchyma was semiautomatically extracted from consecutive MDCT images. Three‐dimensional, volume‐rendered images of the portal vein and hepatic vein were generated from the late arterial or portal phase data using the automatic algorithm of the software. Three‐dimensional images of the portal vein, hepatic vein, and liver parenchyma were reconstructed individually and then overlapped to create integrated 3D images. The vascular perfusion area of the tertiary branch of the right anterior portal vein was calculated using software, and the ramification pattern of the right anterior portal vein was evaluated.

### 2.4 Definitions and study endpoints

**FIGURE 1:**
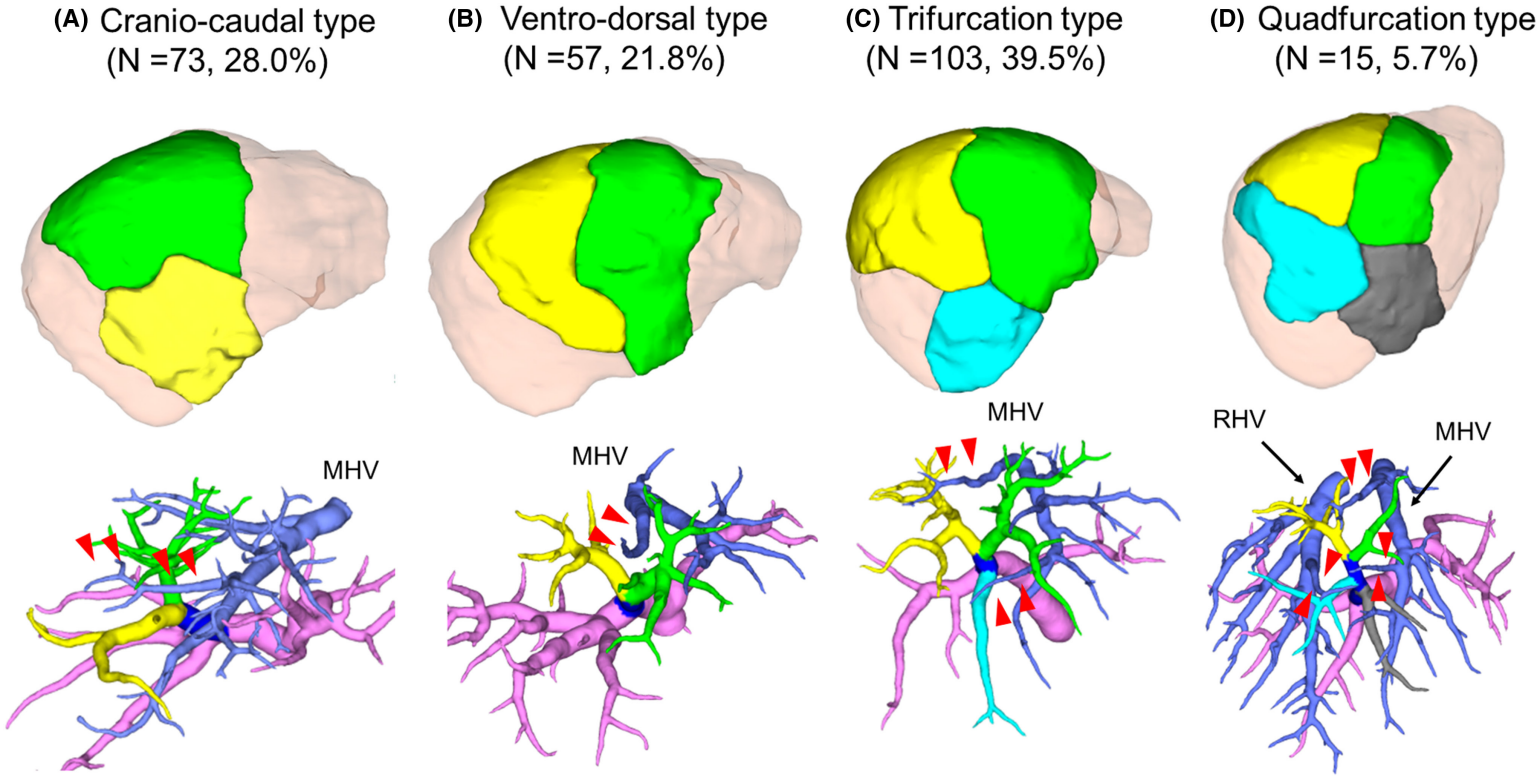
Classification based on the portal perfusion area in right anterior sector. The right anterior sector was divided into four types. A: The craniocaudal type is divided into cranial (green) and caudal (yellow) areas. B: The ventrodorsal type is divided into ventral (green) and dorsal (yellow) areas. C: The trifurcation type is divided into cranioventral (green), craniodorsal (yellow), and caudal (light blue) areas. D: The quadfurcation type is divided into cranioventral (green), craniodorsal (yellow), caudal‐ventral (light blue), and caudal‐dorsal (gray) areas. The right anterior portal vein (second‐order portal branches) is shown in blue. A representative case of the hepatic veins (arrowheads) across the boundary of the areas in each type. MHV, middle hepatic vein; RHV, right hepatic vein

We defined the configuration of the right anterior sector into four types according to the portal venous perfusion area (Figure 1). The definitions of each type are as follows: the craniocaudal type is when the tertiary portal branch of the right anterior sector dominates the cranial and caudal sides and the branch of the middle hepatic vein across the boundary (Figure 1A); the ventrodorsal type is when the tertiary portal branch of the right anterior sector dominates the ventral and dorsal sides and the first branch of the middle or right hepatic vein across the boundary (Figure 1B); the trifurcation and quadfurcation types are when the tertiary portal branch of the right anterior sector dominates the cranioventral/craniodorsal/caudal side and cranioventral/craniodorsal/caudal‐ventral/caudal‐dorsal side, respectively. Some caudal branches were thin; therefore, we defined them as the quadfurcation type when the volume of ventral and dorsal branches on the caudal area were similar. There is a branch of the middle or right hepatic veins at the boundary of each area (Figure 1C,D). The caudal branches are defined as branches running toward the caudal side from the direction of the anterior portal vein. Additionally, the PV8c branch was evaluated according to the following definition based on descriptions in previous reports by Kumon^15^, ^22^ and Trinh Van‐Minh et al^13^ “The portal venous branch that independently branches off the cranial root of the anterior sector portal vein toward the diaphragm and distributes in the area surrounded by the right and middle hepatic veins."

## 3 RESULTS

### 3.1 Classification based on the portal perfusion area in the right anterior sector

In total, 261 patients were evaluated in this study (Figure 1). The craniocaudal, ventrodorsal, trifurcation, and quadfurcation types were found in 130 (49.8%), 57 (21.8%), 103 (39.5%), and 15 (5.7%) patients, respectively (Figure 1). The remaining 13 (5.0%) patients did not have the above four types (unclassified type). Furthermore, the hepatic veins that cross the boundary of the areas were observed in each type.

### 3.2 Ramification pattern of the tertiary portal branch of the right anterior sector

**FIGURE 2:**
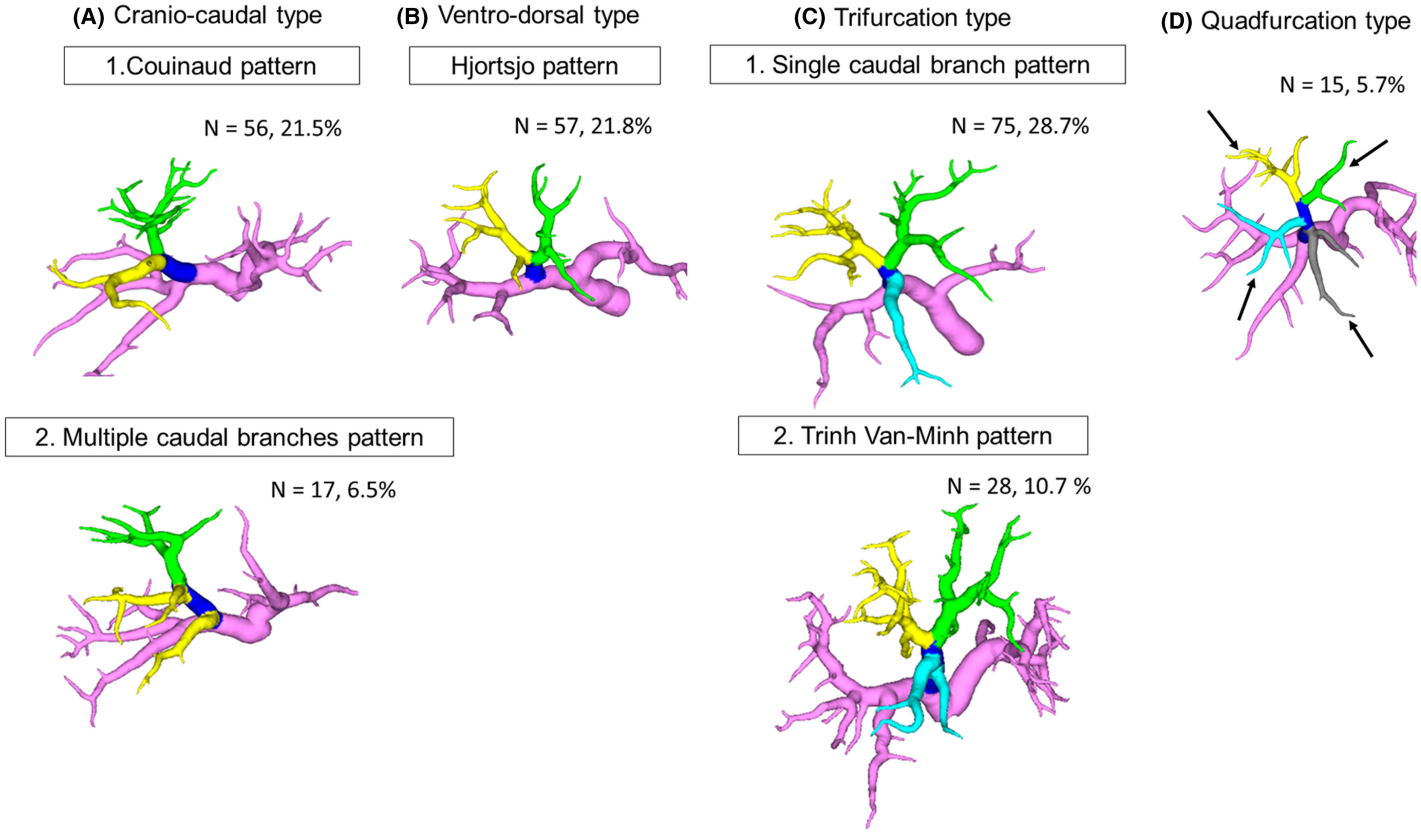
Ramification pattern of the tertiary portal branch of the right anterior sector by each type. A: The craniocaudal type is subdivided into bifurcation and multiple caudal branches patterns. The bifurcation pattern (A‐1) is when the right anterior portal vein bifurcates into the cranial and caudal branches (Couinaud pattern). The multiple caudal branches pattern (A‐2) is when there are multiple caudal branches on the tertiary portal branch. B: The ventrodorsal type is the only bifurcation pattern (Hjortsjo pattern). C: The trifurcation type is subdivided into the single caudal branch pattern and the Trinh Van‐Minh patterns. The single caudal branch pattern (C‐1) is present when one caudal branch ramifies independently or simultaneously with the ventral and dorsal branches from the anterior portal vein. The Trinh Van‐Minh pattern (C‐2) is present when there are two thick caudal branches. D: The quadfurcation type is present when the ventral and dorsal branches of the caudal side ramifies independently or simultaneously with cranial branches from the right anterior portal vein

Next we evaluated the ramification pattern of the tertiary branch of the right anterior sector for each type (Figure 2). The craniocaudal type was further divided into two patterns (Figure 2A): Couinaud pattern (56 of 261, 21.5%), the right anterior portal vein bifurcates into the cranial and caudal branches (Figure 2A‐1); and multiple caudal branches pattern (17 of 261, 6.5%), there are multiple caudal branches (usually two to three) on the tertiary portal branch (Figure 2A‐2).

The ventrodorsal type had only one pattern (Figure 2B): in the Hjortsjo pattern, the right anterior portal vein bifurcates into the ventral and dorsal branches. The trifurcation type was divided into two patterns (Figure 2C) including the single caudal branch pattern (75 of 261, 28.7%), and the Trinh Van‐Minh pattern (28 of 261, 10.7%). The single caudal branch pattern was defined as the case in which one caudal branch ramified independently or simultaneously with ventral and dorsal branches from an anterior portal vein (Figure 2C‐1). Trinh Van‐Minh et al divided the right anterior sector into three subsegments and reported that there were two thick caudal branches.^13^ Therefore, we defined these patterns as the Trinh Van‐Minh pattern (Figure 2C‐2).

The quadfurcation type included only one pattern (Figure 2D): ventral and dorsal branches of the caudal side ramified independently or simultaneously with cranial branches from the right anterior portal vein. A summary of the classification and ramification patterns is shown in Table 1.

**TABLE 1:** Summary of classification and ramification patterns in the right anterior sector (N = 261)

Classification of types	Ramification patterns	Cases	Proportion
Craniocaudal type		73	28.0%
	*Couinaud pattern*	*56*	*(21.5%)*
	*Multiple caudal branches pattern*	*17*	*(6.5%)*
Ventrodorsal type	*(Hjortsjo pattern)*	57	21.8%
Trifurcation type		103	39.5%
	*Single caudal branch pattern*	*75*	*(28.7%)*
	*Trinh Van‐minh pattern*	*28*	*(10.7%)*
Quadfurcation type		15	5.7%
Unclassified type		13	5.0%

### 3.3 Ramification pattern of the unclassified type

**FIGURE 3:**
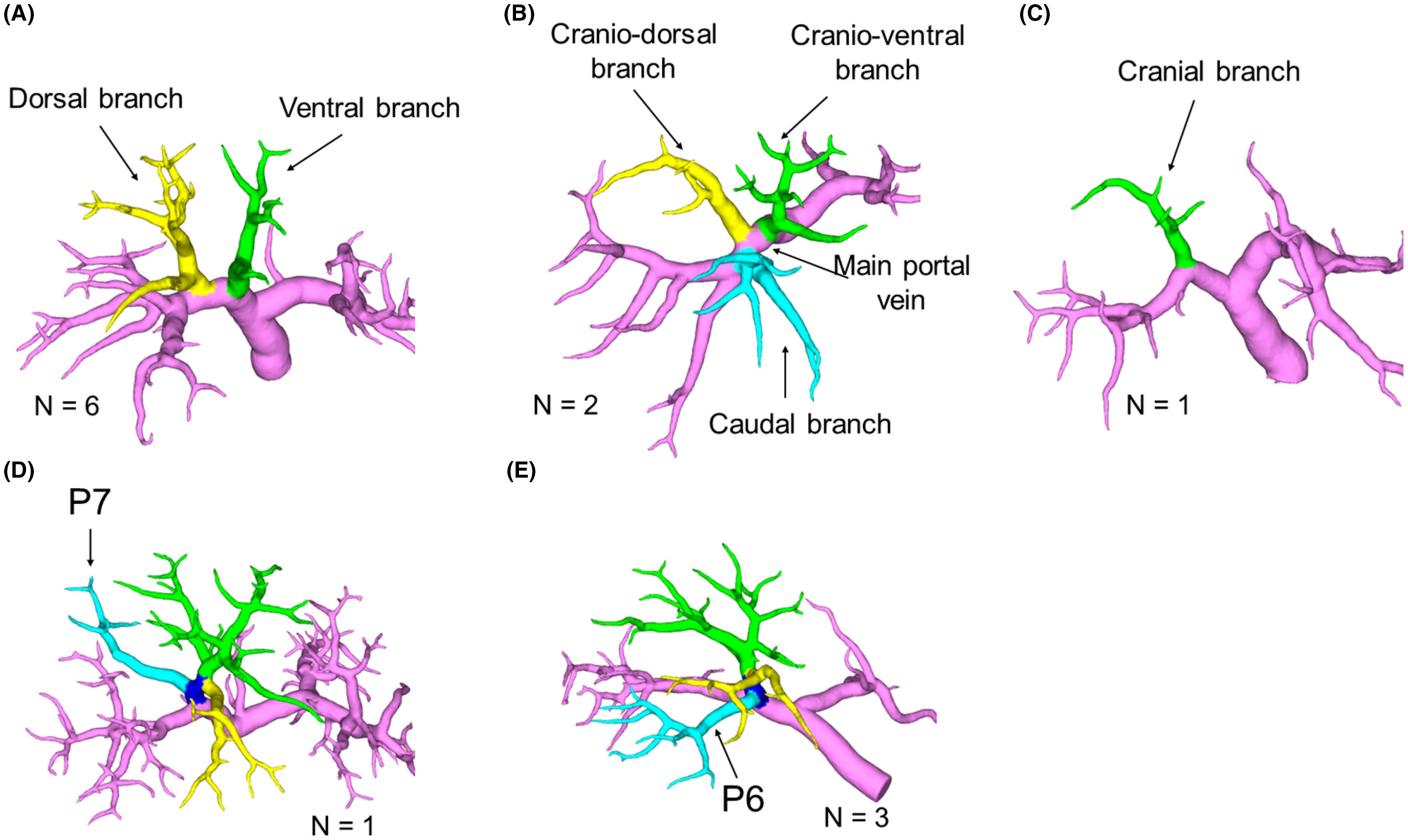
Ramification pattern of the unclassified type. A: The ventral (green) and dorsal (yellow) branches are ramified directly from the right first‐order portal vein independently. B: The cranioventral (green), craniodorsal (yellow), and caudal (light blue) branches are ramified directly from the right first‐order portal vein independently. C: The right anterior portal vein perfuses only the cranial area (green). D: The posterior branch (P7, light blue) is ramified from the right anterior portal vein. E: The posterior branch (P6, light blue) is ramified from the right anterior portal vein. P7, portal vein of segment VII; P6, portal vein of segment VI

Thirteen patients did not have the above four types. The ramification patterns of the unclassified types are shown in Figure 3. Six patients had no definite right anterior portal vein, and the ventral and dorsal branches were ramified from the right portal vein independently (Figure 3A). Two patients also had no definite right anterior portal vein, and cranioventral, craniodorsal, and caudal branches ramified from the right portal vein independently (Figure 3B). One patient had only a cranial branch (portal vein of segment VIII) (Figure 3C). Another patient had a posterior branch (portal vein of segment VII: P7) that was ramified from the right anterior portal vein (Figure 3D). Furthermore, three patients had a posterior branch (portal vein of segment VI: P6) that ramified directly from the right anterior portal vein (Figure 3E).

### 3.4 Identification of the PV8c branch and frequency by 3D images

**FIGURE 4:**
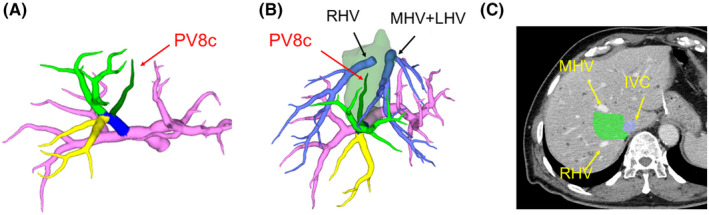
Identification of the PV8c branch. A: Representative case of the PV8c branch. The PV8c branch ramifies from the contralateral side of the caudal branch of the right anterior portal vein. B: The perfusion area of the PV8c branch is calculated by 3D‐CT image analysis. The PV8c branch has perfused the territory surrounded by the roots of the right and middle hepatic veins. This representative case is classified as the craniocaudal type (bifurcation pattern). C: The axial CT image of the perfusion area of PV8c (green area). IVC, inferior vena cava; LHV, left hepatic vein; MHV, middle hepatic vein; RHV, right hepatic vein

The PV8c branch was identified on the cranial side of the root of the right anterior portal vein. The representative PV8c branch is shown in Figure 4A. This representative case was classified into the craniocaudal type (Couinaud pattern), and the PV8c branch ramified from the contralateral side of the caudal branch of the portal vein root and dominated the territory surrounded by the roots of the right and middle hepatic veins by analysis of the perfusion area using 3D images and axial images of CT (Figure 4B,C). The frequency of PV8c branch identification was 140 of 261 patients (53.6%), mean volume of the PV8c area was 41.6 mL (range 7–89 mL), and mean proportion of the PV8c area in the whole liver volume was 3.4% (0.8%–6.8%). The summary of frequency, mean volume, and proportion of the whole liver volume of the PV8c branch in each type is shown in Table 2. The ventrodorsal type had a lower frequency of PV8c compared with other types. The volume and proportion of the whole liver volume of the PV8c was similar in each type.

**TABLE 2:** Summary of PV8c branch in each type

Classification of types	Frequency of PV8c (%)	Volume of PV8c perfusion area (ml)	Proportion of PV8c area(%)
Craniocaudal type (n = 73)	44/73 (60.3%)	40.2 (7–89)	3.3%
Ventrodorsal type (n = 57)	22/57 (38.6%)	42.9 (27–69)	3.7%
Trifurcation type (n = 103)	57/103 (55.3%)	44.5 (11–76)	3.5%
Quadfurcation type (n = 15)	11/15(73.3%)	33.0 (15–56)	2.5%

## 4 DISCUSSION

Although the right anterior sector was divided into the superior and inferior areas of the main portal arch by Couinaud^1^, ^3^ and Healey and Schroy,^2^ and Couinaud described the boundary between segments V and VIII as the right portal vein trunk of the hepatic hilum,^1^, ^9^ the reason why the boundary of the anterior sector is the hepatic hilum has not been shown. The segmentation of the right anterior sector is still controversial because of the lack of a definite anatomical landmark between segments V and VIII. However, after rediscovery of the Hjortsjo anatomical classification,^14^ some researchers proposed that the right anterior sector should be divided into ventral and dorsal segments.^25^, ^26^ In particular, Cho et al emphasized that the right anterior portal vein bifurcated into the ventral and dorsal branches in all cases, and the border between the ventral and dorsal segments is the so‐called anterior fissure vein.^5^, ^26^ However, not all the cases reported by Cho et al are true, considering our results. This ventrodorsal segmentation is better for parenchymal‐preserving hepatectomy when the functional reserve is limited.^5^, ^27^ However, Mikami and Trinh Van‐Minh et al, who evaluated anatomy using cadaveric livers and liver casts, respectively, proposed another alternative, with subsegmentation of the right anterior sector into three segments.^12^, ^13^ Although there are many variations in the branches of the portal vein of the anterior sector, Trinh Van‐Minh et al classified them into cranioventral, craniodorsal, and caudal branches as a typical ramification pattern.^13^ In the cranial region, they added an accessory inconstant portal vein branch that is identical to the PV8c branch proposed by Kumon.^15^, ^22^ Further, in the caudal region of the anterior sector, Trinh Van‐Minh et al presented two thick portal vein branches and two thin portal vein branches that distribute to the gallbladder bed.^13^ Kogure et al also described the existence of these thin caudal branches running toward the gallbladder bed, and the number of these branches was usually two, although it varied from one to four branches.^14^ These three craniocaudal, ventrodorsal, and trifurcate anatomical segmentations would presumably represent the main anatomical variations of the portal branches of the right anterior sector, as reported in recent 3D imaging studies.^18^, ^20^ In the present study, we classified the right anterior sector mainly into four types: craniocaudal, ventrodorsal, trifurcation, and quadfurcation, according to the classification by Kurimoto et al.^5^ Kurimoto et al classified the right anterior sector mainly into three types: craniocaudal, ventrodorsal, and multiple types, and subclassified the multiple types into trifurcation and quadfurcation types. In our series, the trifurcation type was dominant (103 of 261, 39.5%). However, other studies using 3D analysis have reported that the dominant type of classification varies. Some authors described that the craniocaudal type was dominant (Kobayashi et al: 53 of 100 [53%], Kurimoto et al: 184 of 370 [49.7%]),^5^, ^18^ whereas another study reported that the ventrodorsal type was dominant (Cho et al: 60 of 60 [100%]).^19^ Although it is difficult to clearly explain such a discrepancy among the studies, the reason may be that it is difficult to definitively distinguish craniocaudal and trifurcation types. We focused on the ramification of the third‐order portal vein and evaluated branches that can be drawn by 3D imaging one by one, while some studies excluded branches that are estimated to be less than 10% of the whole liver volume^5^ and defined the trifurcation type as the case in which three branches ramify at the same point simultaneously.^18^ This may also explain the discrepancy between the results. Our study included independent branches, not only those ramifying the same point (Figure 2C). Considering this point, the frequency of the craniocaudal type plus trifurcation type (176 of 261, 67.5%) in our study was similar to that of previous reports in which the craniocaudal type plus trifurcation type was dominant.^5^, ^18^ On the other hand, the quadfurcation type had a lower frequency (5.7%) compared with other types. Based on the low frequency of the quadfurcation type, it can be speculated that the quadfurcation type depicted in our study is only a subtype of the trifurcation type, in which the caudal branches are well developed. However, we cannot disregard the fact that a certain number of cases have ventral and dorsal branches of the caudal side with equally large volumes, suggesting that the quadfurcation type is an independent type. Therefore, we defined the quadfurcation type as an independent type, rather than a subtype of the trifurcation type. Next, we subclassified each type into six patterns according to the ramification form of the tertiary portal branch. There are few reports of a detailed ramification analysis of the portal vein using 3D imaging; mainly, it was reported in studies using percutaneous transhepatic portography.^16^, ^17^ Inoue et al classified eight basic branching patterns of the right anterior portal vein, and this classification was conducted according to the point where the caudal branch ramified.^17^ Further, Ichida et al classified ramification pattern of P5 into nine patterns using magnetic resonance imaging.^24^ In our study, caudal branches were typically recognized as multiple branches that ramified from various points of the right anterior portal vein, and our subclassification depended on the ramification pattern of the caudal branches. These results suggest that the complexity of the caudal branches of the right anterior portal vein may be one cause of confusion in the classification of the right anterior sector.

Recently, portal vein branching of the human liver has been increasingly investigated using 3D imaging of CT. It has been emphasized that classifying the portal vein ramification pattern in the anterior sector preoperatively is important for parenchymal‐preserving hepatectomies, especially for cases in which the functional reserve was limited.^5^, ^6^, ^27^ Kurimoto et al reported that, of the 270 hepatectomies of HCC, parenchymal‐preserving hepatectomy of the right anterior sector was performed in 32 cases, the ventral region was presented in 14 cases, and the dorsal region was presented in 18 cases.^5^ Furthermore, Kogure et al reported an HCC case of the paracaval portion in the caudate lobe with poor functional reserve; central hepatectomy was performed using a parenchymal‐sparing approach to resect segments I, IV, and VIII.^6^ In this case, the liver parenchymal of the anterior sector was transected along the border between the ventral and dorsal regions, and the dorsal region of segment VIII was preserved.^6^ Thus, an understanding of the ramification pattern of the anterior portal veins is essential for considering parenchymal‐preserving hepatectomy, especially in cases with poor functional reserve.

Thirteen patients were classified as having the unclassified type. Although the types in which the so‐called tertiary portal branch ramified directly from the right portal vein (Figure 3A,B) can be considered as the ventrodorsal or trifurcation type from the perspective of the perfusion area, respectively, we considered it as an unclassified type because of the lack of a definite right anterior portal vein. The types where the branches of the posterior sector ramified from the right anterior portal vein (Figure 3D,E) were rare (4 of 261, 1.5%). However, these types require careful attention during surgery when planning to resect the tumor‐bearing Glissonean area for HCC treatment and using the Glissonean pedicle transection method via the hepatic hilum approach.^28^

We evaluated a unique, characteristic portal branch of the right anterior portal vein, which was described as the medial branch of segment VIII (PV8c) by Kumon, and an accessory inconstant portal venous branch by Trinh Van‐Minh et al in a cast study of the caudate lobe.^13^, ^15^ Kumon reported that PV8c was recognized in 12 of 18 corrosion liver casts, and emphasized it as an important branch considering the ventral right‐sided boundary of the paracaval portion of the caudate lobe.^22^ Takayasu et al^16^ and Inoue et al^17^ have also reported branches equivalent to PV8c in studies of portography, and the frequencies were 34 of 173 cases and 16 of 28 cases, respectively. Further, Mise et al reported the frequency of P8med, which is equivalent to PV8c as 52 of 107 cases in the study of 3D‐CT analysis.^23^ In our study, the frequency of the PV8c branch was 53.6% (140 of 261), which is similar to that reported by Kumon, Takayasu et al, and Mise et al. The present study is the largest series compared with previous studies. When considering the right‐sided boundary, especially the right‐sided ventrolateral border, of the paracaval portion of the caudate lobe, it is still controversial whether the PV8c branch belongs to the branch of the right anterior sector or caudate lobe. Ishiyama et al described a branch ramified from the right posterior portal vein that perfuses the right‐sided dorsolateral border of the paracaval portion (dl‐PCP) by analysis using liver casts,^29^ and they proposed that dl‐PCP should be removed when performing complete resection of the caudate lobe. When considering the PV8c branch in the right anterior sector as the counterpart of dl‐PCP in the posterior sector, resection of the PV8c perfusion area should be considered to obtain the surgical margin of the right‐sided ventrolateral border of the paracaval portion (vl‐PCP) during complete resection of the caudate lobe. Takayama et al^30^ reported 43 cases of total resection of the caudate lobe and indicated that the posterior surfaces of the right and left hepatic vein were completely exposed as the resecting plane. This indicates that the area of the PV8c is included in the resection area. Even if the PV8c belong to the branch of the anterior sector portal vein, it is technically difficult to resect an entire caudate lobe without including areas of the PV8c. The PV8c perfusion area is only 3.4% of the total liver volume; therefore, it is reasonable to include this area in the resection of the total caudate lobe. Thus, although PV8c is an anatomically important branch in the right anterior sector, it is possible that this branch is excluded in 3D image analysis because the proportion of the PV8c area in the whole liver volume is small (mean, 3.4%). However, the PV8c branch is meaningful in clinical practice, and it is important to keep in mind the existence of the PV8c branch when considering the right‐sided boundary of the caudate lobe.^30^, ^31^ When the PV8c branch, an anatomical landmark of the right‐sided boundary of the caudate lobe, is absent, the counterstaining technique reported by Takayama et al is effective in determining the right‐sided boundary of the caudate lobe intraoperatively.^32^ Moreover, with recent advances in laparoscopic hepatectomy, a hepatic vein‐guided approach for resection of segment VIII has been proposed.^33^, ^34^ In this procedure, liver parenchymal transection was initiated from the root of the middle hepatic vein, and reached the Glissonean pedicle of the anterior sector, especially segment VIII on the right side of the middle hepatic vein. Considering that PV8c ramifies from the cranial aspect of the root of anterior portal vein, it might be a landmark to reach the Glissonean pedicle of the anterior sector in cases where PV8c is present.

The current study has a couple of limitations. First, the 3D image analysis depends on the contrast conditions. Although contrast‐enhanced CT has been performed under the same conditions, some patients show poor visualization of vessels, such as the portal vein and hepatic vein, due to differences in hemodynamics. In such cases, it is difficult to accurately evaluate the ramifications of the tertiary portal branch. This is the most significant difference from the study of cadavers and liver casts. Second, in our series patients had hepatobiliary and pancreatic diseases. Although patients with liver cirrhosis and vascular invasion of tumors were excluded, it is better to analyze healthy patients, such as donors of living transplantation, ideally.

In conclusion, we classified the right anterior sector into four types according to the perfusion area of the tertiary portal branch, and subclassified each type into six patterns according to the ramification form. In particular, the ramification patterns of caudal branches varied, and we showed the complexity of the caudal branches. Furthermore, we evaluated the details of PV8c, such as frequency, perfusion area, and proportion of the whole liver volume of the PV8c branch for the first‐time using 3D image analysis.

## DISCLOSURE

The authors declare no conflicts of interest for this article.
